# Host Response against Virus Infection in an Insect: Bidensovirus Infection Effect on Silkworm (*Bombyx mori*)

**DOI:** 10.3390/antiox10040522

**Published:** 2021-03-27

**Authors:** Katsuhiko Ito, Kangayam M. Ponnuvel, Keiko Kadono-Okuda

**Affiliations:** 1 Department of Science of Biological Production, Graduate School of Agriculture, Tokyo University of Agriculture and Technology, 3-5-8 Saiwai-cho, Fuchu, Tokyo 183-8509, Japan; 2 Silkworm Genomics Division, Seribiotech Research Laboratory, Carmelaram-Post, Kodathi, Bangalore 560035, India; kmpvel@gmail.com; 3 Department of Research Promotion, Institute of Agrobiological Sciences, National Agriculture and Food Research Organization, Tsukuba, Ibaraki 305-8634, Japan; kadono@affrc.go.jp

**Keywords:** bidensovirus infection, insect immune response, silkworm, sericulture industry, silk production

## Abstract

Silk cocoons obtained from silkworms are the primary source of commercial silk, making the silkworm an economically important insect. However, the silk industry suffers significant losses due to various virus infections. *Bombyx mori* bidensovirus (BmBDV) is one of the pathogens that cause flacherie disease in silkworms. Most silkworm strains die after BmBDV infection. However, certain silkworm strains show resistance to the virus, which is determined by a single recessive gene, *nsd-2*. The +*^nsd-2^* gene (allele of *nsd-2*; the susceptibility gene) encodes a putative amino acid transporter expressed only in the insect’s midgut, where BmBDV can infect, suggesting that this membrane protein may function as a receptor for BmBDV. Interestingly, the expression analysis revealed no changes in the +*^nsd-2^* gene expression levels in virus-uninfected silkworms, whereas the gene expression drastically decreased in the virus-infected silkworm. This condition indicates that the host factor’s expression, the putative virus receptor, is affected by BmBDV infection. It has recently been reported that the expression levels of some host genes encoding cuticle, antioxidant, and immune response-related proteins were significantly regulated by BmBDV infection. In this review, we discuss the host response against virus infection based on our knowledge and long-term research experience in this field.

## 1. Introduction

*Bombyx mori* has been bred in captivity for around 5000 years, and it is now a completely domesticated species of the silkworm [1]. The silkworm is an economically important insect that makes silk cocoons. Therefore, sericulture is an important industry that was promoted in various Asian and European countries. The most severe threat in the sericulture industry is the lack of silkworm disease control. Many pathogens such as viruses, bacteria, fungi, and microsporidia have been found to infect silkworms, making it challenging to control the spread of these diseases. Remarkably, the economic damage caused by viruses (i.e., nucleopolyhedrovirus (NPV), cypovirus (CPV), infectious flacherie virus, and densovirus (DNV)] is so significant that the sericulture industry’s disease control is critical to keep the silk production. Many studies have been carried out to elucidate the interaction between the silkworm and the viruses, and a variety of molecules and pathways involved in the silkworm’s immune responses to viruses, such as antimicrobial peptides, prophenoloxidase-activating system, apoptosis, reactive oxygen species (ROS), small RNA, and related molecules, have been identified [2]. Considering this background, we focused on the DNV’s infection mechanism and host responses to the virus, and we were successful in isolating a vital host factor (putative virus receptor) for the first time in the insect virus research field [3,4]. In this review, we would like to discuss the following items: (i) the silkworm’s resistance mechanism against the DNV infection; (ii) the relationships between a putative virus receptor and DNV infection; (iii) the host genes whose expression levels fluctuate due to DNV infection.

## 2. Characterization of *Bombyx mori* Densovirus and Bidensovirus

*Bombyx mori* densovirus (BmDNV) is a pathogen that causes flacherie disease in the silkworm. The BmDNV was previously divided into type-1 (BmDNV-1) and type-2 (BmDNV-2 and -Z) based on differences in their virulence toward silkworms, serological characteristics, and genomic structures [5,6,7] (Table 1). BmDNV-1 was classified into the *Iteravirus* genus, while BmDNV-2 and -Z were classified into the *Bidensovirus* genus, in the *Densovirinae* subfamily or parvovirus-like viruses. At present, BmDNV-1 has been reassigned to the *Iteradensovirus* genus, and has been renamed BmDV [8] (Table 1). On the other hand, BmDNV-2 and -Z were excluded from the *Parvoviridae* family because of their bipartite genome structure and a DNA polymerase motif within their genomes. These viruses have been reassigned to the *Bidensovirus* genus, *Bidnaviridae* family, and are designated as *B. mori* bidensovirus (BmBDV) [8] (Table 1). To date, one isolate of BmDV has been reported: namely, the Ina isolate (Japan; BmDNV-1) [9] (Table 1). On the other hand, four BmBDV isolates have been reported: namely, Yamanashi and Saku isolate (Japan; BmDNV-2) [10], and China and Zhenjiang isolate (China; BmDNV-3, BmDNV-Z) [11] (Table 1). A new BmBDV isolate was recently identified from the Indian sericulture industry, named the Indian isolate [12] (Table 1).

## 3. Pathogenicity of BmDV and BmBDV

The BmDV and the BmBDV multiply only in the columnar cells’ nuclei in the larval midgut epithelium, and no pathological changes were observed in the other tissues [9,10,13] (Table 1). The midgut epithelium’s histopathological studies from silkworms infected with BmDV showed hypertrophic nuclei in the columnar cells markedly stained with the Feulgen reaction of methyl green [13]. The degenerated columnar cells are eventually liberated into the midgut’s lumen [13,14]. In the BmBDV infection, almost identical features were observed in the midgut [10]. However, the course of the BmDV infection was significantly different compared with that of the BmBDV infection. Larvae infected with BmDV show body flaccidity as the infection’s most significant sign, and die about seven days post-infection (DPI) [14,15]. On the other hand, the BmBDV infection is accompanied by no appreciable external symptoms until 6–7 DPI, and the time of death varies between 10 and 20 DPI [15,16,17]. After getting infected, a few infected larvae manage to pupate [15]. These results indicate that BmDV infection is acute, whereas BmBDV infection is chronic [15] (Table 1).

## 4. Silkworm Resistance to BmDV and BmBDV

**Figure 1:**
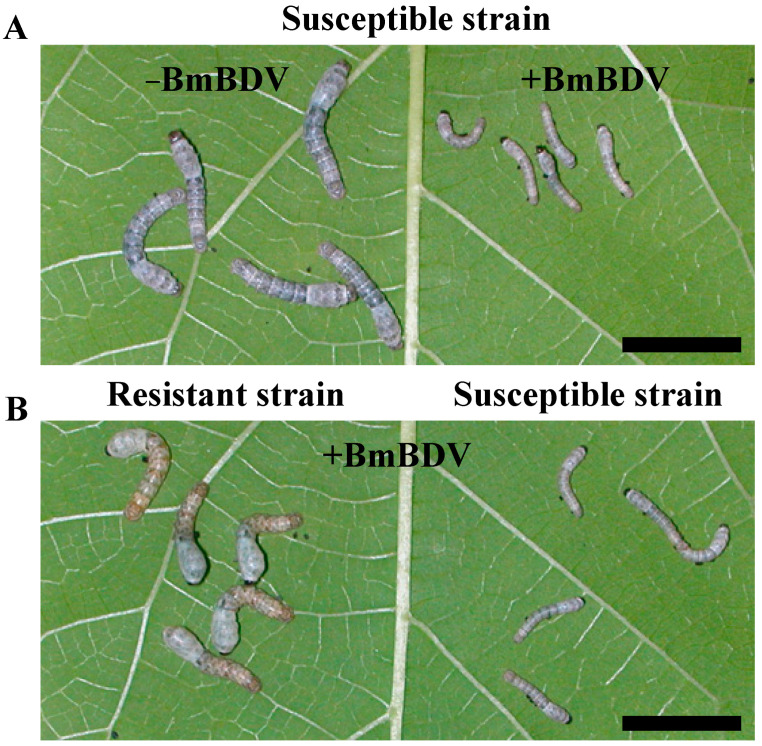
The effect of BmBDV infection on silkworm larvae. (**A**) Comparison of the BmBDV uninfected and infected susceptible silkworm strain at nine days post infection (DPI). Left and right show the virus-uninfected and virus-infected silkworms, respectively. (**B**) Comparison of BmBDV infected resistant and susceptible silkworm strains at nine DPI. Both are the virus-infected silkworm. Scale bar, 10 mm.

In general, there are two ways by which insects resist infection/disease. They are “Infection resistance,” in which it is difficult for the virus to establish infection in the insects, and “Disease resistance,” in which it is difficult to propagate the infection and spread the infection even after infected [5]. By such a resistance mechanism, the host suppresses the virus infection’s progression and damage. However, the silkworms’ resistances to BmDV and BmBDV are different from the above; they are a unique phenomenon called “Complete resistance” in which insects are completely unaffected by viruses, even when the virus inoculation’s amount and frequency increases (Figure 1). Interestingly, such resistances were found in certain silkworm strains, and their offspring inherited the resistance trait [5,6,18] (Figure 1). Thus, the data suggest that the resistance gene is transferred from generation to generation.

Four unrelated BmDV or BmBDV infection’s resistance genes, such as *nonsusceptibility gene against BmDNV-1* (*nsd-1*) [18], *Non-infectious gene against BmDNV-1* (*Nid-1*) [19], *nonsusceptibility gene against BmDNV-2* (*nsd-2*) [20], and *nonsusceptibility gene against Zhenjiang (China) strain of BmDNV* (*nsd-Z*) [21] were reported thus far (Table 1). These virus resistance genes were unidentified for a long time. However, positional cloning using the *B. mori* genome information successfully identified two genes, *nsd-1* and *nsd-2*, from the silkworm’s genome [3,4].

## 5. Identification of the Gene *nsd-2*, Responsible for Resistance to BmBDV Infection

Based on the *nsd-2* gene’s locus information on *B. mori*’s chromosome 17 [20], positional cloning was performed to identify the candidate region of *nsd-2* [3]. The one candidate gene was identified through positional cloning to be delimited to an approximately 400 kb-long region on chromosome 17. The candidate gene encodes a putative transporter protein, and there are significant differences noted in the gene’s sequence between resistant and susceptible strains. The candidate gene derived from the +*^nsd-2^* gene (allele of *nsd-2*, the susceptibility gene) encodes a protein containing a putative 12-pass transmembrane domain, whereas that from the *nsd-2* gene (the resistance gene) encodes a truncated membrane protein that contains only the first three-pass transmembrane domain. The expression analysis also revealed that the candidate gene was expressed only in the midgut where BmBDV can infect and replicate (Table 1). These results strongly suggest that this gene is the best candidate for *nsd-2* [3].

**Figure 2:**
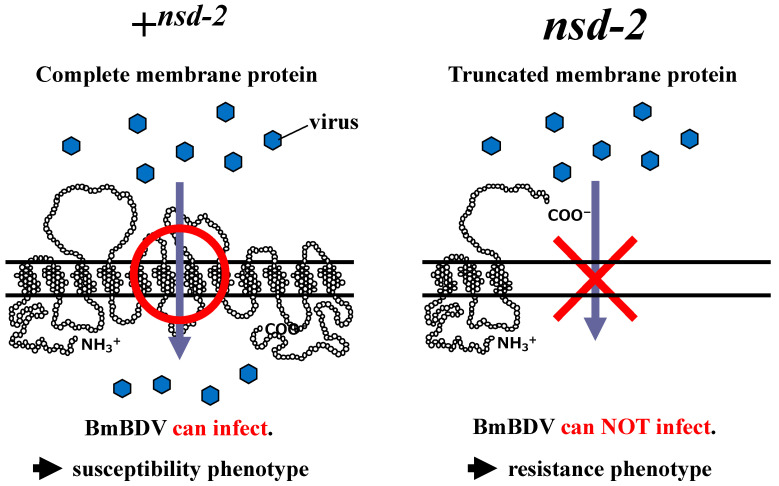
Relationship between BmBDV infectivity and virus susceptibility (+*^nsd-2^*)/ resistance (*nsd-2*) gene products. The secondary structures of the NSD-2 protein derived from the +*^nsd-2^* gene (left) and the *nsd-2* gene (right) are based on the topology prediction method, SOSUI (http://harrier.nagahama-i-bio.ac.jp/sosui/ accessed on 17 December 2007).

To verify whether the candidate gene is responsible for virus resistance in the *nsd-2* mutants, we generated a transgenic silkworm in which the +*^nsd-2^* candidate gene (the susceptibility gene) was introduced into a resistant strain (homozygous *nsd-2* gene genotype) using the GAL4-UAS system [22]. After virus inoculation, the transgenic silkworm showed a remarkable susceptibility phenotype, suggesting that the candidate gene is *nsd-2* itself, the virus resistance gene, and the complete membrane protein expressed by the allele, +*^nsd-2^*, is required for infection by BmBDV [3] (Figure 2).

## 6. Putative Function of NSD-2

The European Molecular Biology Laboratory’s accession number AB365597 with 12 transmembrane domains (+*^nsd-2^*) and the resistance type (*nsd-2*) of which only the three first transmembrane domains are intact, revealed that the NSD-2 protein expressed by +*^nsd-2^* gene showed high homology with two amino acid transporters of the tobacco hornworm, *Manduca sexta* (GenBank accession nos. AF006063 with 77% and AF013963 with 76% identities) by NCBI-BLAST (https://blast.ncbi.nlm.nih.gov/Blast.cgi/ accessed on 17 December 2007) [23,24]. These amino acid transporters function as a K^+^-coupled amino acid transporter [23] and Na^+^- or K^+^-activated nutrient amino acid transporter [24]. Whether the NSD-2 protein functions as a transporter or not is still unknown, but it may have a role similar to that of *M. sexta*-derived transporters because of its high sequence homology. Interestingly, the resistant strains possessing a large deletion of this transporter grow normally under appropriate conditions. This observation strongly suggests that the NSD-2 protein is not essential for the silkworm’s development.

During the positional cloning, at the genomic locus near *nsd-2*, we found one gene, *AK378309*, that also encodes a putative transporter but has no relationship to virus resistance because there is no sequence difference between both resistant and susceptible strains [3]. This gene’s deduced amino acid sequence shows a relatively high sequence identity (68.2%) with an NSD-2 protein expressed by the +*^nsd-2^* gene. Therefore, the protein expressed by this gene might function as an amino acid transporter instead of NSD-2.

## 7. The NSD-2 Protein’s Location on Midgut and Relationship with BmBDV Infectivity

**Figure 3:**
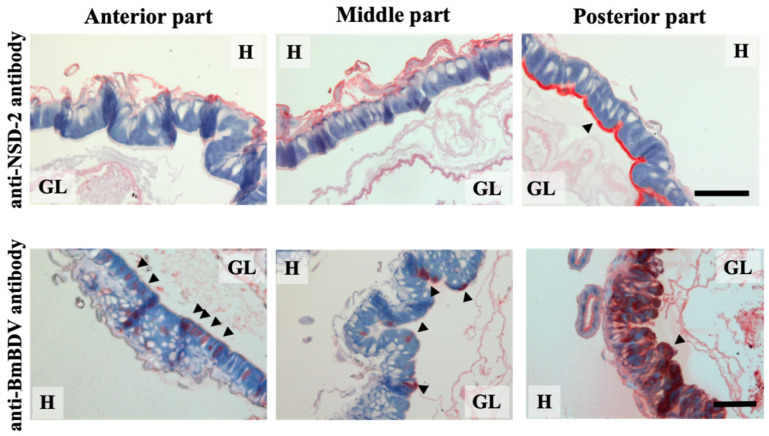
The NSD-2 and the BmBDV protein’s location in the different parts of the midgut. The upper and lower panels show the immunofluorescence analysis using an anti-NDS-2 antibody and anti-BmBDV antibody, respectively. Paraffin section prepared from the midgut of larvae from the fifth instar day three or four were developed with the ABC Kit and 3-Amino-9-ethylcarbazole solution, and the nuclei were counterstained with hematoxylin. Red-colored regions and arrowheads show the NSD-2 and BmBDV protein’s locations. GL, gut lumen; H, hemolymph. Scale bar, 100 μm. This figure is a partial modification of Ito et al. [17].

To better understand BmBDV and NSD-2 protein’s relationship, we performed an immunohistochemical analysis in the midgut [17]. Although Nakagaki et al. [25] reported that the mode of BmBDV replication varies depending on the part of the midgut, we investigated the loci expressing both proteins in different parts of the insect’s midgut: the anterior, middle, and posterior portions. The immunohistochemical analysis showed that the NSD-2 protein expressed by the +*^nsd-2^* gene, which expressed the complete membrane protein, was strongly expressed in the posterior part of the midgut and was less detected in both anterior and middle portions (Figure 3). On the other hand, the BmBDV protein was detected in all parts of the midgut (Figure 3). However, there was a remarkable difference among the parts of the midgut. Hypertrophic nuclei were observed in the columnar cells of the posterior part of the virus-infected midgut (Figure 3). In the anterior part of the midgut, some of the nuclei were hypertrophied. However, the damage to anterior part of the midgut was not as substantial as the posterior part of the midgut (Figure 3). In the middle part of the midgut, the hypertrophic nuclei were observed in only a few columnar cells, and associated cellular degradation was not detected (Figure 3). These results suggest that the BmBDV infectivity may depend on the NSD-2 protein’s expression level and that virus infection can be caused even by a very faint NSD-2 expression [17].

## 8. Influence of the Silkworm by BmBDV

**Figure 4:**
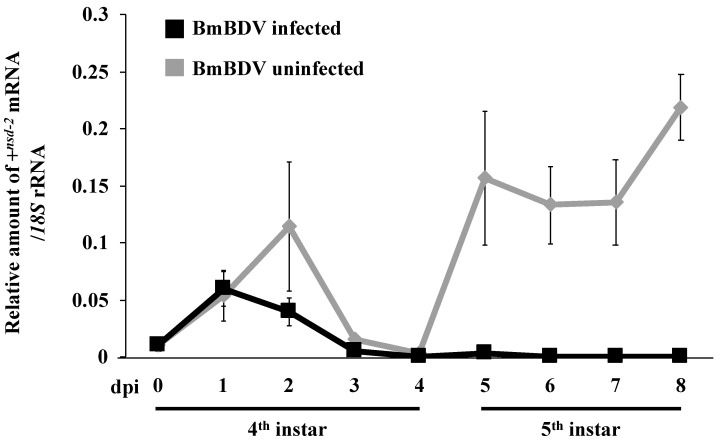
The time course of +*^nsd-2^* mRNA expression in the midgut. Black and gray bars show the result of virus-infected and virus-uninfected silkworms, respectively. Four DPI indicates a molting stage. We measured these transcripts’ expression patterns using cDNA prepared from the posterior part of the midgut. We used *B. mori*’s *18S* rRNA as an endogenous control. Data are represented by mean ± standard error (*n* = 3). This figure is a partial modification of Ito et al. [26].

The +*^nsd-2^* gene encodes a complete membrane protein containing a putative 12-pass transmembrane domain expressed in the midgut where the BmBDV can infect [3,17] (Figure 2). Therefore, we speculated that the NSD-2 protein expressed by the +*^nsd-2^* gene might act as a receptor for BmBDV. We compared the +*^nsd-2^*gene’s expression pattern in the midgut of the virus-uninfected and virus-infected silkworm to investigate the relationship between BmBDV and NSD-2 [26]. In the case of the virus-uninfected silkworm, a quantitative RT-PCR revealed that the +*^nsd-2^*gene was clearly expressed at each stage, except during the end of instar including a molt when new columnar cells are generated from regenerative cells (Figure 4) [3]. In contrast, in the case of virus infection, the +*^nsd-2^*gene’s expression level drastically decreased from two DPI (Figure 4).

**Figure 5:**
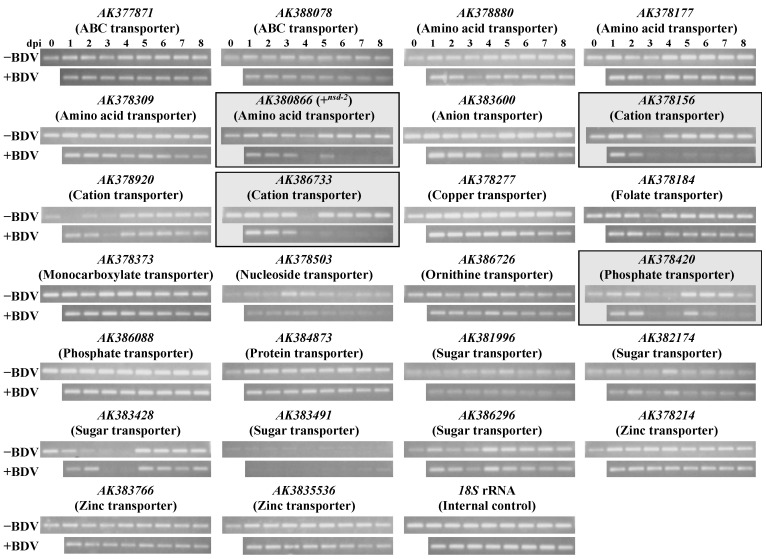
The expression pattern of 26 genes encoding transporter proteins in the midgut of virus-uninfected and virus-infected silkworm larvae. The upper and lower panels show the results of virus-uninfected and virus-infected silkworms, respectively. *B. mori 18S* rRNA was used as an internal control. The expression levels of four genes: *AK380866* (+*^nsd-2^*; amino acid transporter), *AK378156* (cation transporter), *AK386733* (cation transporter), and *AK378420* (phosphate transporter) were significantly decreased by virus infection. This figure is a partial modification of Ito et al. [26].

We examined the expression level of genes encoding other transporters in the midgut after virus infection [26] to investigate whether the decrease in expression levels caused by virus propagation is exhibited only by the +*^nsd-2^* gene. For this examination, we performed gene screening using the KAIKObase, which is an integrated silkworm genome database (http://sgp.dna.affrc.go.jp/KAIKObase/ accessed on 7 May 2018) [27,28], and selected 26 genes encoding transporters that are expressed in the midgut: two genes for the ABC transporter; four genes (including +*^nsd-2^*) for the amino acid transporter; one gene for the anion transporter; three genes for the cation transporter; one gene for the copper transporter; one gene for the folate transporter; one gene for the monocarboxylate transporter; one gene for the nucleoside transporter; one gene for the ornithine transporter; two genes for the phosphate transporter; one gene for the protein transporter; five genes for the sugar transporter; and three genes for the zinc transporter. Next, we performed RT-PCR analyses with the primer sets corresponding to the 26 transporter genes. The RT-PCR analyses revealed no difference in the expression levels in most genes between the virus-uninfected and virus-infected silkworms (Figure 5). However, only three genes’ expression levels were significantly decreased by virus infection (Figure 5), showing a similar decrease in the expression level as the decrease by the +*^nsd-2^* gene (*AK380866*, amino acid transporter), *AK378156* (cation transporter), *AK386733* (cation transporter), and *AK378420* (phosphate transporter). These results cumulatively suggest that the four transporters (including the +*^nsd-2^*gene) are affected by BmBDV infection [26].

## 9. Host Response by BmBDV

Liu et al. [29] performed gene expression analyses after virus infection to investigate the BmBDV effects on the signaling pathways associated with the insect’s innate immunity. In their analyses, the genes they evaluated were *BmSTAT*, *spatzle-1* (*Bmspz-1*), *peptidoglycan-recognition protein LB* (*BmPGRP-LB*), *peptidoglycan-recognition protein LE* (*BmPGRP-LE*), *argonaute 2* (*Bmago2*), and *dicer-2* (*Bmdcr2*). The analyzed signaling pathways associated with the insect’s innate immunity were involved in the Janus kinase (JAK)/signal transducer and activator of transcription (STAT), Toll, immune deficiency (Imd), and miRNA-mediated pathways. As a result, only the JAK/STAT pathway could be active when challenged with BmBDV, suggesting that this pathway is used as the silkworm’s BmBDV defense [29].

A transcriptome analysis of the silkworm’s immune response at the early stage of BmBDV infection was recently reported by Sun et al. [30]. According to this report, a gene ontology analysis of differentially expressed genes revealed that genes responsible for the cuticle’s structural constituents, antioxidant, and immune system processes, such as apoptosis and autophagy, were significantly up- and down-regulated after BmBDV infection [30]. In the cuticle protein, two genes, *BmorCPR23* and *BmorCPR44*, classified as secreted cuticle proteins (CPs) with the Rebers and Riddiford motif, were induced by BmBDV [30]. Yang et al. [31] have reported that CP from the pacific white shrimp is involved in white spot syndrome virus invasion as a co-receptor, suggesting that the two gene products might be involved in the invasion process of BmBDV. Antioxidant-related genes encoding catalase (Cat), superoxide reductase (SOR), and superoxide dismutase (SOD) were induced by BmBDV [30]. Generally, virus infection causes the host to produce excessive ROS leading to oxidative stress [30,32,33,34]. Therefore, these genes might be involved in eliminating excessive ROS caused by viruses. Moreover, the apoptosis-related genes such as a *fas-associated protein with death domain* and *high-temperature-regulated A2* and autophagy-related genes responsible for the immune system were also induced by BmBDV [30,35,36,37]. These results strongly suggested that apoptotic signaling pathways and autophagy are activated for antivirus after BmBDV infection.

## 10. Conclusions

**Figure 6:**
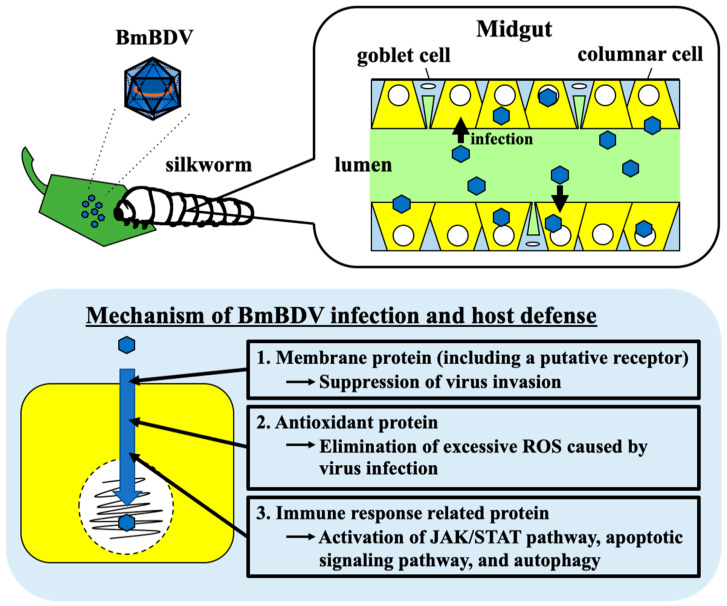
The BmBDV infection mechanism and host defense.

The BmBDV is one of the major silkworms’ pathogens [12,38]. The virus isolate has been found in Japan, China, and India, and their characteristics and pathogenicity to silkworms have been studied so far [10,11,12]. However, there were many unclear points about the mechanism of interaction between silkworms and BmBDV. We succeeded in identifying the essential host genes, *nsd-2* and +*^nsd-2^*, responsible for BmBDV resistance and susceptibility, respectively [3,17]. We also found that BmBDV infection alters these genes’ expression levels [26]. Other groups recently performed the silkworms’ transcriptome analysis after BmBDV infection and revealed that the expression levels of some genes encoding cuticle, antioxidant, and immune response-related proteins were significantly up- and down-regulated [29,30]. Therefore, some of the relationship between silkworms and BmBDV became clearer (Figure 6).

There is a piece of evidence that a virus infection regulates some host genes involved in innate immunity, such as BmNPV, BmCPV, and BmBDV [29,30,39,40,41,42,43,44,45]. However, the virus’ receptor gene’s regulation has not been reported yet. Our result indicated that the expression level of the +*^nsd-2^* gene encoding the putative receptor for BmBDV was significantly decreased by virus infection (Figure 4) [26]. This result provided new findings on the interaction between host and virus in *B. mori*. Although we still do not have clear answers about the advantages and disadvantages between host and virus in the decreased expression levels of the +*^nsd-2^*gene, we can posit that this may be an infection protective mechanism of the host against viruses. By controlling the receptor’s expression level, the host cells may reduce the damage caused by virus infection.

In the examination of the expression level of genes encoding other transporters in the midgut after virus infection, a decrease in the expression level was observed in only four of the 26 genes encoding an amino acid transporter (*AK380866*, +*^nsd-2^*), a cation transporter (*AK378156* and *AK386733*), and a phosphate transporter (*AK378420*) (Figure 5) [26]. There are four genes (*AK378880*, *AK378177*, *AK378309*, and *AK380866*) encoding an amino acid transporter. However, only the +*^nsd-2^* gene expression level decreased with virus infection (Figure 5). Interestingly, the expression level of *AK378309*, which shows a high amino acid sequence homology with the +*^nsd-2^* gene (68.2%) and the same number of transmembrane domains [3] did not decrease after virus infection (Figure 5) [26]. According to Sun et al. [30], some genes encoding CPs, which might be a candidate virus co-receptor, were significantly up-regulated. These results suggest that the effects of virus infection were not caused by many membrane proteins expressed in the midgut but only by specific membrane proteins. However, it is not yet clear which characteristics of these proteins are essential. Therefore, further analysis will be required to detect the interaction between BmBDV and these proteins.

Sun et al. [30] also reported that the antioxidant genes *BmCat*, *BmSOD3*, *BmSOR4*, and *peroxisomes genes peroxin-5*, *peroxin-10*, *peroxin-13* were significantly up-regulated after BmBDV infection. These results indicate that this virus causes silkworms to produce excessive ROS, which induces an antioxidant mechanism to prevent oxidative stress and triggers anti-viral peroxisomes against BmBDV, thereby protecting the host [30]. Moreover, Liu et al. [29] and Sun et al. [30] revealed that BmBDV significantly induced JAK/STAT-, apoptosis-, and autophagy-related genes responsible for the immune system. These results also provide insights into the mechanisms of BmBDV infection and host defense.

Thus, recent studies have revealed host-derived genes that are altered by BmBDV infection. We expect that further analysis of these genes will elucidate the host stress response mechanism due to insect virus infection.

## Figures and Tables

**Table 1:** Salient features of *Bombyx mori* densovirus (BmDV) and *B. mori* bidensovirus (BmBDV).

Virus Type	*Bombyx mori* Densovirus (BmDV)	*Bombyx mori* Bidensovirus (BmBDV)
Previous classification	*Bombyx mori* densovirus type-1 (BmDNV-1)	*Bombyx mori* densovirus type-2 (BmDNV-2)
Isolates	Ina isolate (Japan)	Yamanashi isolate (Japan), Saku isolate (Japan), China isolate (China), Zhenjiang isolate (China), Indian isolate (India)
Family/Genus	*Parvoviridae/Iteradensovirus*	*Bidnaviridae/Bidensovirus*
Virion Size	20 nm	24 nm
Genome Topology	Linear	Linear
Genome Type	ssDNA	Segmented ssDNA
Genome Size	5.0 kb	6 kb; 6.5 kb
Number of ORFs	3	6 or 7
Pathogenicity	acute	chronic
Virus Affected Tissue	Midgut columnar cell	Midgut columnar cell *
Resistance Gene	*Nid-1* and *nsd-1*	*nsd-2* and *nsd-z*

* Chinese isolates of BmBDV infect the goblet cells of midgut epithelium during the late stage of infection.

## Data Availability

The datasets generated from this study supporting our finding are available from the corresponding author on reasonable request.
